# Hypothalamic-Pituitary-Adrenal (HPA) Axis Functioning in Overtraining Syndrome: Findings from Endocrine and Metabolic Responses on Overtraining Syndrome (EROS)—EROS-HPA Axis

**DOI:** 10.1186/s40798-017-0113-0

**Published:** 2017-12-08

**Authors:** Flavio A. Cadegiani, Claudio E. Kater

**Affiliations:** Adrenal and Hypertension Unit, Division of Endocrinology and Metabolism, Department of Medicine, Escola Paulista de Medicina, Universidade Federal de São Paulo (EPM/UNIFESP), R. Pedro de Toledo 781 – 13th floor, São Paulo, SP 04039-032 Brazil

**Keywords:** Overtraining syndrome, Sports endocrinology, Hypothalamus-pituitary-adrenal axis, Hormones

## Abstract

**Background:**

Overtraining syndrome (OTS) results from excessive training load without adequate recovery and leads to decreased performance and fatigue. The pathophysiology of OTS in athletes is not fully understood, which makes accurate diagnosis difficult. Previous studies indicate that alterations in the hypothalamus-pituitary-adrenal (HPA) axis may be responsible for OTS; however, the data is not conclusive. This study aimed to compare, through gold standard and exercise-independent tests, the response of the HPA axis in OTS-affected athletes (OTS group) to healthy physically active subjects (ATL group) and healthy non-active subjects (NCS group).

**Methods:**

Selected subjects were evaluated for cortisol response to a 250-μg cosyntropin stimulation test (CST), cortisol and adrenocorticotropic hormone (ACTH) responses during an insulin tolerance test (ITT), and salivary cortisol rhythm (SCR).

**Results:**

A total of 51 subjects were included (OTS, *n* = 14; ATL, *n* = 25; and NCS, *n* = 12). Cortisol response in the CST was similar among the three groups. Conversely, mean cortisol response during an ITT was significantly higher in ATL (21.7 μg/dL; increase = 9.2 μg/dL) compared to OTS (17.9 μg/dL; 6.3 μg/dL) and NCS (16.9 μg/dL; 6.0 μg/dL) (*p* ≤ 0.001; *p* = 0.01). Likewise, median ACTH response during an ITT was significantly higher in ATL (91.4 pg/mL; increase = 45.1 pg/mL) compared to OTS (30.3 pg/mL; 9.7 pg/mL) and NCS (51.4 pg/mL; 38.0 pg/mL) (*p* = 0.006; *p* = 0.004). For SCR, mean cortisol 30 min after awakening was significantly higher in ATL (500 ng/dL) compared to OTS (323 ng/dL) and NCS (393 ng/dL) (*p* = 0.004). We identified the following cutoffs that could help exclude or confirm OTS: cortisol level at 30 min after awakening (exclusion = > 530 ng/dL); cortisol response to ITT (exclusion = > 20.5 μg/dL; confirmation = < 17 μg/dL or increase < 9.5 μg/dL); and ACTH response (exclusion = > 106 pg/mL or increase > 70 pg/mL; confirmation = < 35 pg/mL and increase < 14.5 pg/mL).

**Conclusion:**

The findings of the present study showed that healthy athletes disclose adaptions to exercises that helped improve sport-specific performance, whereas this sort of hormonal conditioning was at least partially lost in OTS, which may explain the decrease in performance in OTS.

## Key-points

- The hypothalamus-pituitary-adrenal (HPA) axis response to ITT are exacerbated in healthy athletes, compared to sedentary healthy subjects. ITT may be a tool to evaluate whether the athlete is well-conditioned and to predict performance, once the exacerbation of the HPA axis responses may play an important role in the progressive improvement in sports performance.
- There are intrinsic dysfunctions of the HPA axis response to a stress situation in OTS-affected athletes, compared to healthy athletes, in an independent way from exercise-induced stimulation; the dysfunctions of the HPA axis are located in the hypothalamus and the pituitary, and not the adrenals. In case an athlete is suspected for OTS, an ITT stimulation test may be performed. In the absence of confounding diseases, blunted cortisol and ACTH responses most likely confirm the diagnosis of OTS, with accurate cutoffs.
- Two new concepts were unprecedentedly demonstrated by the study. The first new concept is that physical activity, at least moderate-to-intense, elicits conditioning effects of hormonal responses to stimulation that goes beyond exercise, which we called as “hormonal conditioning of the athlete”. Besides helping explain the improvement in the sports performance, the novel conditioning process found by our study may be the missing link for the understanding of the underlying mechanisms of the improvement observed in several responses to harmful situations, such as infections, neoplasms, traumas, inflammations, and psychiatric conditions that are observed in athletes, and which were not fully understood so far. The second concept is that whereas healthy athletes seem to present hormonal conditioning adaptions, those affected by overtraining seem to have impaired or maladapted hormonal conditioning, as over-trained athletes have a blunted optimized hormone response to stress that seem to be acquired by athletes; as a sort of deconditioning process, which indicates that the decreased performance and the reduced time-to-fatigue observed during OTS; these two key features of OTS, not yet fully understood, may be at least partly explained by the present findings.

Despite these unprecedented findings, further studies are recommended to confirm our results.

## Background

The combination of excessive training load and lack of adequate recovery can lead to a decrease in sport-specific performance associated with fatigue, termed overtraining syndrome (OTS). Similar states that are related to OTS include functional overreaching (FOR) and non-functional overreaching (NFOR), which differ in terms of the duration of the signs and symptoms and in performance after recovery [1].

Improper recovery, low calorie intake, social problems, and excessive progression in training volume and intensity are the key triggers of OTS and correlate states. An inhospitable environment resulted from the inability to recover from intense energy demands induced an extensive dysfunctional adaption (maladaptation) with consequent abnormal responses of multiple markers [1].

As OTS and related states can affect 15–60% of elite athletes [1], and there are a growing number of high performance athletes, understanding the pathophysiology of OTS is essential [1–3]. As OTS is a diagnosis of exclusion, it is important to screen for inflammatory, metabolic, hormonal, psychiatric, and infectious conditions that could be the primary reason of decrease in sports performance [1–4].

Among some proposed biomarkers for diagnosis of OTS, impaired hormonal responses to stressful tests induced by maximal exercise have been reported [1–9], although further reproduction of the findings is recommended. With regard to the likely cause of the impaired hormonal responses, chronic exposure to stress could decrease the responsiveness of hypothalamic pituitary adrenal (HPA) axis [1, 4, 7–12]. Other alterations of the HPA axis were related to fatigue states and could be helpful for the diagnosis of OTS, such as a decreased cortisol awakening response (CAR) [13–17], which is an expected physiological increase in cortisol levels upon waking, and altered salivary cortisol rhythm [13, 14]. A decrease in CAR does not necessarily indicate impaired HPA axis function, but more likely worsened sleep, which is one of the features of OTS [1, 3, 12–30].

The impaired hormonal response to exercise-induced stress reported in previous studies [7–9] may result from impaired signaling from the musculoskeletal and cardiovascular systems to the HPA axis, rather than a decreased responsiveness of the glands. Moreover, in a recent systematic review [3], we learned that none of the previous studies were performed with gold standard functional tests, which are standardized to identify hormones at the primary site of the dysfunction caused by OTS (that is, the changes are not due to a lack of external signaling).

In this study, we performed a complete evaluation of the HPA axis at different levels by employing gold standard stimulation tests that are independent of exercises to ensure the hormone findings are the primary responses. The present study also aimed to correlate other cortisol findings [18–27] with OTS and to find new criteria for OTS.

## Methods

A detailed description of the materials and methods and the raw data can be found elsewhere [31], but the inclusion and exclusion criteria, study design, questionnaire, and tests performed in this study are described below.

### Inclusion and Exclusion Criteria

Subjects were recruited through social media (Facebook and Instagram) and contacted by the main researcher (FAC) and were required to be male, have present body mass index (BMI) of 20.0–32.9 kg/m^2^ (for athletes) and 20.0–30.0 kg/m^2^ (for non-athletes), and be 18–50 years old, who do not present previous psychiatric disorders, do not use any centrally acting drugs, or have not used any hormonal therapy in the previous 6 months. This was phase 1 of the inclusion criteria.

A minimum amount of physical activity was required from athletes, as specified in the EROS study methodology manuscript [31]. In particular, a minimum of four sessions and 300 min of moderate-to-intensive training per week was required, at a training level typical for professional competition, and at least 6 months of continuous training. In addition, the subject must be referred to as an athlete by a professional coach.

Volunteers suspected of OTS were required to disclose underperformance of at least 10% of reduction of the previous performance, as confirmed by a sports-related coach that regularly follows the athlete, unexplained by conditions that could lead to reduction in performance including infections (particularly Epstein-Barr), inflammation, overt hormonal dysfunctions (that would be the primary cause of the decreased performance), and psychosocial or psychiatric conditions, whose exclusion of the confounding conditions was performed by the main researcher (FAC). For inclusion in the OTS group, athletes must show persistent fatigue, an increased sense of effort in trainings that required less effort prior to the OTS, worsening in sleep quality (this criteria was not obligatory), and fulfill the recommended protocol for diagnosis of OTS proposed by the most recent guidelines on OTS [1].

Remaining subjects then underwent biochemical examination (phase 2 of the inclusion process). Subjects were to show levels within the normal range to be included in this study, in order to prevent confounding diagnosis.

### Design of the Study

All selected subjects signed a written informed consent for participation in the study, approved by the ethics committee of the Federal University of São Paulo.

In the EROS-HPA axis arm of the study, we evaluated peripheral and central components of the HPA axis (primary or peripheral: adrenal; central: pituitary and hypothalamus), with cosyntropin stimulation test, that directly evaluates adrenal responses to synthetic ACTH [32], insulin tolerance test (ITT), which evaluates the integrity of the HPA axis [33], and salivary cortisol rhythm, to identify patterns of the circadian rhythm of the cortisol [13–17].

### Questionnaire

After the selection criteria, an initial private interview about training patterns was performed with the selected athletes (sedentary subjects were not assessed at this moment), as part of the EROS study (for all arms). The interview included questions regarding training intensity (self-evaluation and evaluation by a professional coach, on a scale from 0 to 10 compared to athletes of the same level of training) and time since starting training. Other questions asked in the interview included whether they worked or studied besides training, and if they did, how many hours they worked or studied per day (on average); self-referred libido (on a scale from 0 to 10 compared to libido 1 year prior to the questionnaire); details of food ingestion each day in the 7 days prior to the questionnaire (including number of calories per kilogram per day, grams of carbohydrates, proteins and fats per kilogram per day); and sleeping patterns (average duration of sleep, self-referred sleep quality on a scale from 0 to 10, and presence or absence of initial insomnia and terminal insomnia). For all athletes affected by OTS, questions regarding the average number of days to overcome the underperformance state and changes in sensitivity to heat or to coldness or in sleep quality was performed.

### Testing of the HPA Axis

A sequence of tests was then performed in all subjects, including the cosyntropin stimulation test (CST), the insulin tolerance test (ITT), and the salivary cortisol rhythm (SCR).

### Cosyntropin Stimulation Test (CST)

The cosyntropin (synthetic adrenocorticotropic hormone [ACTH]) stimulation test is performed with a synthetic ACTH in high doses (250 μg) in order to directly stimulate the adrenal glands.

For the CST, blood was collected (time 0) from the antecubital vein of all subjects at 8.00 AM, after 30 min of resting and 8 h of fasting, and 250 μg of cosyntropin was infused. Blood was then collected at 30 min (time 1) and 60 min (time 2) for analysis of cortisol response (that is, the cortisol increase in absolute levels [μg/dL] in response to the infusion of 250 μg of cosyntropin stimulation).

### Insulin Tolerance Test (ITT)

We used a gold standard ITT to evaluate the intrinsic responsiveness and integrity of the HPA axis, since a normal response required absence of dysfunctions in all levels (hypothalamus, pituitary, and adrenals) of the HPA axis. With a normal CST response, any abnormality found in the ITT is located either in the hypothalamus or in the pituitary. Unlike exercise stimulation tests, whose hormone responses depend on external neuromuscular and cardiovascular signaling, an impaired hormone response to ITT means the dysfunction is truly located in the HPA axis.

Subjects performed the ITT 48 h after the CST, following the same protocol of fasting, arrival time, and resting period prior to the beginning of the ITT. Blood was initially collected (time 0), a dose of 0.1 IU/kg of regular insulin was infused intravenously and new blood was collected during hypoglycemia (time 1) and 30 min after (time 2). After the blood collection during hypoglycemia, 10 mL of 50% glucose solution was given intravenously, and high-glycemic index food was offered ad libitum. At all times, cortisol (μg/dL), ACTH (pg/mL), and glucose (mg/dL) were assessed, and absolute ACTH/cortisol ratio was calculated.

Due to the risk of ITT-induced severe hypoglycemia (unconsciousness), three doses of subcutaneous glucagon were available (GlucaGen HypoKit, 1 μg, NovoNordisk), as well as syringes containing 20 mL of 50% glucose solution and an automated external defibrillator (AED).

### Salivary Cortisol Rhythm

After stimulation tests, we assessed salivary cortisol rhythm (SCR) as an attempt to reproduce previous findings in fatigue-related states studies [3, 15], as a potential marker of fatigue.

From 2 to 7 days after ITT, saliva was collected at the time of awakening, at 30 min after awakening, at 4 PM, and at 11 PM, and specific recommendations were provided. Saliva samples were collected by the subjects themselves, using laboratory kits provided by the researcher (FAC).

All hormones of the present study (salivary cortisol, serum cortisol, and serum ACTH) were analyzed by specific electrochemiluminescence assays using specific commercial kits at a laboratory, whereas serum glucose levels were analyzed by an enzymatic assay of hexokinase. Importantly, the 8 AM serum cortisol was collected at the same time, while salivary cortisol was collected at the awakening moment and 30 min later, regardless if, and for this reason, salivary and serum cortisol levels are not comparable.

### Statistical Analysis

Statistical analysis was performed using IBM SPSS statistics 24 software (IBM, USA). Each parameter was compared among the three groups (OTS, ATL, and NCS), and pairwise tests were performed between OTS and ATL, OTS and NCS, and ATL and NCS, whenever *p* < 0.05. As none of the variables depend on other variables, main and interaction effects were not evaluated (cortisol levels at a certain time do not reflect the ACTH levels at the same time).

Criteria for normal distribution was evaluated using Kolgomogorov–Sminorv test. Whenever normal distribution criteria were met, one-way ANOVA tests were performed, whereas when results were not normally distributed, Kruskal-Wallis tests (nonparametric ANOVA tests) were performed. Adjusted Dunn’s test, Dunnett’s T3, and Tukey analyses were performed when differences were statistically significant between the three groups (*p* < 0.05), according to the normality criteria.

## Results

### Baseline Characteristics and Training Patterns of the Study Participants

From the 146 initially recruited subjects, 51 subjects were included in the study (34.2%), and divided in three different groups, of OTS-affected athletes (OTS group; *n* = 14), healthy athletes (ATL group; *n* = 25), and healthy but physically non-active control subjects (normal control subjects [NCS]; *n* = 12). All athletes performed both resistive and endurance exercises, and all the training patterns were similar between OTS and ATL. All OTS were classified as OTS (and not FOR or NFOR) due to their prolonged recovery time.

Regarding the baseline characteristics, the average age (OTS = 30.6 years, ATL = 32.7 years, and NCS = 33.2 years) and average BMI (OTS = 26.7 kg/m^2^, ATL = 24.9 kg/m^2^, and NCS = 33.2 kg/m^2^) were statistically similar among the groups. In addition, the average number of minutes of training per week (OTS = 574.3 min and ATL = 550.0 min), mean training intensity (OTS = 8.79 and ATL = 8.76, on a scale from 0 to 10), and number of training days per week (OTS = 5.36 days and ATL = 5.46 days) were similar between the two groups of athletes.

### Basal Hormone Levels

As summarized in Tables 1, 2, and 3, basal serum hormone levels of OTS are within the normal range, and similar to ATL and to NCS, as previously observed [1–4, 6–9, 17–29, 34–36].

**Table 1:** Mean (±SD) basal serum cortisol and response to a cosyntropin stimulation test (CST) with 250 μg of synthetic ACTH

Cortisol response to CST (μg/dL)	OTS athletes	Healthy athletes	Sedentary
Basal	13.1 (± 4.1)	12.1 (± 3.2)	12.1 (± 5.7)
30	19.1 (± 1.9)	19.7 (± 2.4)	19.7 (± 3.2)
60′	21.9 (± 2.4)	22.2 (± 2.9)	22.9 (± 4.4)

All comparison/analyses were not significant

SD = Standard deviation

**Table 2:** Mean (±SD) basal serum cortisol and response to insulin tolerance test (ITT)

Cortisol response to ITT (μg/dL)	OTS athletes (OTS) (*n* = 14)	Healthy athletes (ATL) (*n* = 25)	Non-active subjects (NCS) (*n* = 12)
Basal	11.6 (± 2.5)	12.5 (± 3.1)	10.9 (± 2.8)
During hypoglycemia	12.4^*^ (± 3.3)	15.9^&^ (± 5.3)	11.8 (± 3.1)
30′ after hypoglycemia	17.9^***^ (± 2.9)	21.7^&&&&^ (± 3.1)	16.9 (± 4.1)
Absolute increase from basal to 30 min after hypoglycemia	6.3^**^ (± 2.3)	9.2^&^ (± 3.7)	5.9 (± 3.9)

Differences between OTS and ATL: *p < 0.05; **p < 0.01; ***p < 0.005

Differences between ATL and NCS: ^&^ *p* < 0.05; ^&&&&^ *p* < 0.001

*SD* standard deviation

**Table 3:** Median (95% CI) ACTH response to insulin tolerance test (ITT)

ACTH response to ITT (pg/mL)	OTS athletes (OTS) (*n* = 14)	Healthy athletes (ATL) (*n* = 25)	Non-active subjects (NCS) (*n* = 12)	*p*
Basal	19.6 (11.4–32.9)	18.7 (6.5–37.8)	21.4 (8.7–37.8)	n/s
During hypoglycemia	28.2 (8.4–238.9)	57.8 (7.3–229.5)	29.5 (14.8–191.7)	n/s
30′ after hypoglycemia	30.3^****^ (9.8–93.7)	59.9 (22.1–195.7)	51.4 (22.7–137.5)	*0.006*
Absolute increase	9.7^****^ (−14.4–64.4)	45.1 (22.1–195.7)	38.0 (0.5–108.8)	*0.004*
Percentage increase (%)	52.2	266.2	200.4	– n/a

Differences between OTS and ATL: ****p < 0.001

Level of significance = *p* < 0.05

*CI* confidence interval; *n/s* = non significant; *n/a* = non appliable

### Evaluation of the Adrenal Gland—Cosyntropin Stimulation Test (CST)

As summarized in Table 1, cortisol response to 250 μg cosyntropin stimulation disclosed a normal and comparable responses between OTS, ATL, and NCS. The normal cortisol responses observed in the OTS-affected athletes confirm that adrenals are unimpaired in the OTS [33].

### Evaluation of the HPA Axis—ITT

**Fig. 1:**
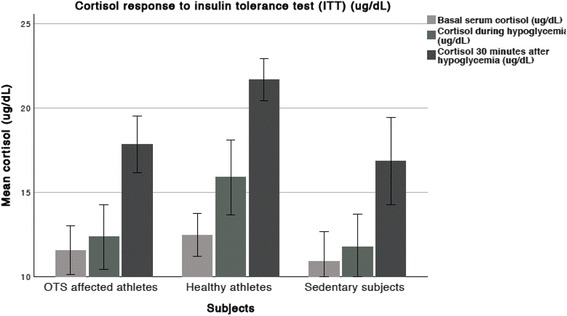
Cortisol response to an ITT

While basal cortisol levels were similar among the three groups, cortisol levels were higher in the ATL during and 30 min after hypoglycemia compared to the OTS and NCS. The mean cortisol increase was higher in the ATL compared to the OTS and NCS (Table 2 and Fig. 1).

At 30 min after hypoglycemia, seven (85.7%) OTS, five (20%) ATL, and nine (75%) NCS showed cortisol levels < 18 μg/dL. A cortisol peak of < 17 μg/dL in response to hypoglycemia was reached by four (28.6%) OTS, none of ATL, and eight (66.7%) NCS. Cortisol levels of > 20.5 μg/dL in response to hypoglycemia were found in none of OTS, 15 (60%) ATL, and three (25%) NCS. A cutoff of 19.1 μg/dL disclosed the highest accuracy (84.6%) to distinct ATL from OTS, although was not precise for neither confirmation nor exclusion of OTS. Furthermore, a cortisol increase of > 9.5 μg/dL was not observed in any OTS, but was found in 15 (60%) ATL and two (16.7%) NCS. A cortisol response of < 17.0 μg/dL at 30 min after the peak of hypoglycemia in the ITT seems to be a reliable cutoff to help confirm the diagnosis of OTS with a likely high positive predictive value when evaluated in athletes. Conversely, a cortisol response of > 20.5 μg/dL and a cortisol increase of > 9.5 μg/dL during the ITT seems to have a high negative predictive value (100%) according to our findings. Whereas ATL displayed a prompt cortisol response to hypoglycemia, OTS and NCS exhibited a delayed cortisol response.

**Fig. 2:**
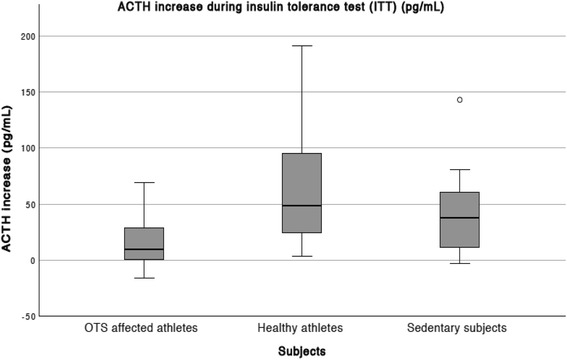
Median and CI (P5-P95) ACTH increase in response to an ITT

**Fig. 3:**
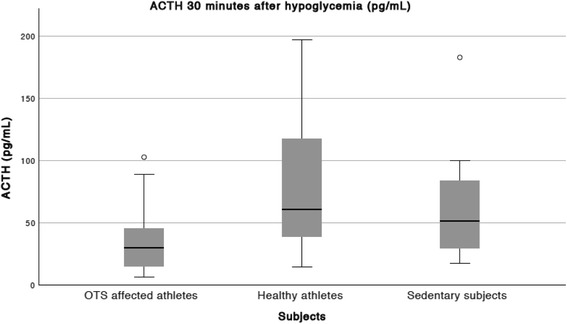
Median and CI (P5-P95) plasma ACTH 30 min after hypoglycemia, in response to an ITT

Basal ACTH and ACTH during hypoglycemia were similar between the three groups (Table 3). However, the late increase of ACTH was higher in the ATL compared to the OTS (Fig. 2). Meanwhile, the median ACTH levels 30 min after hypoglycemia in the NCS were lower than in the ATL and higher than in the OTS, although the differences were not statistically significant, whereas ACTH increase was higher in the ATL than the OTS (Fig. 3).

An ACTH peak of > 46 pg/mL was observed in three OTS (21.4%), in 18 ATL (72%), and in eight NCS (66.7%); there is a cluster of blunted ACTH response to ITT among OTS including 78.6% of subjects. ACTH were < 35 pg/mL in nine (57.1%) OTS, in three (12%) ATL, and in four (33.3%) NCS, with an accuracy of 80% to distinct OTS. Conversely, ACTH peak > 106 pg/mL was observed in only one OTS (an outlier) whereas it was observed in eight ATL (32%).

Two (14.3%) OTS, 14 (56%) ATL, and seven (58.3%) NCS showed a > 34 pg/mL increase in absolute ACTH levels. Meanwhile, no OTS, 10 (40%) ATL, and three (25%) NCS showed a > 70 pg/mL increase in ACTH. The highest accuracy was found for a cutoff of 20 pg/mL (77.0%), although imprecise to determine the absence or presence of OTS.

Based on our results, an ACTH response of > 106 pg/mL seems to be highly predictive of exclusion of OTS, whereas an ACTH response of < 35 pg/mL increases the likelihood of the diagnosis of OTS. Despite the highly variable ACTH increase levels, an increase of > 70 μg/dL could be useful to exclude OTS, whereas blunted ACTH increase was only observed in OTS and NCS.

In a subgroup of the ATL, ACTH and cortisol were also performed 60 min after hypoglycemia, and disclosed lower levels than 30 min, and therefore were not measured in the remaining subjects.

During the ITT, ACTH/cortisol ratio was similar between groups basally and during hypoglycemia, but was significantly lower in the OTS group compared to the ATL (*p* = 0.024) and NCS (*p* = 0.018) groups (Table 4).

**Table 4:** Median (p95 confidence interval) ACTH/cortisol ratios during insulin tolerance test (ITT)

ACTH/cortisol ratio	OTS athletes (OTS) (*n* = 14)	Healthy athletes (ATL) (*n* = 25)	Non-active subjects (NCS) (*n* = 12)	*p*
Basal	184.9 (90.8–332.5)	141.5 (77.4–270.1)	178.0 (91.9–347.3)	n/s
During hypoglycemia	252.3 (77.1–1312.1)	363.5 (111.3–1421.3)	332.9 (122.0–1976.3)	n/s
30′ after hypoglycemia	156.0^*^ (60.4–598.0)	286.2^&^ (96.6–986.5)	321.9 (146.3–723.6)	*0.03*

Differences between OTS and ATL: *p < 0.05

Differences between ATL and NCS: ^&^ *p* < 0.05

*CI* confidence interval

Glucose levels were similar initially and during hypoglycemia between groups, reinforcing the equality of conditions of the ITT and foreclosing differences due to the intensity of hypoglycemia.

### Salivary Cortisol Rhythm (SCR)

**Fig. 4:**
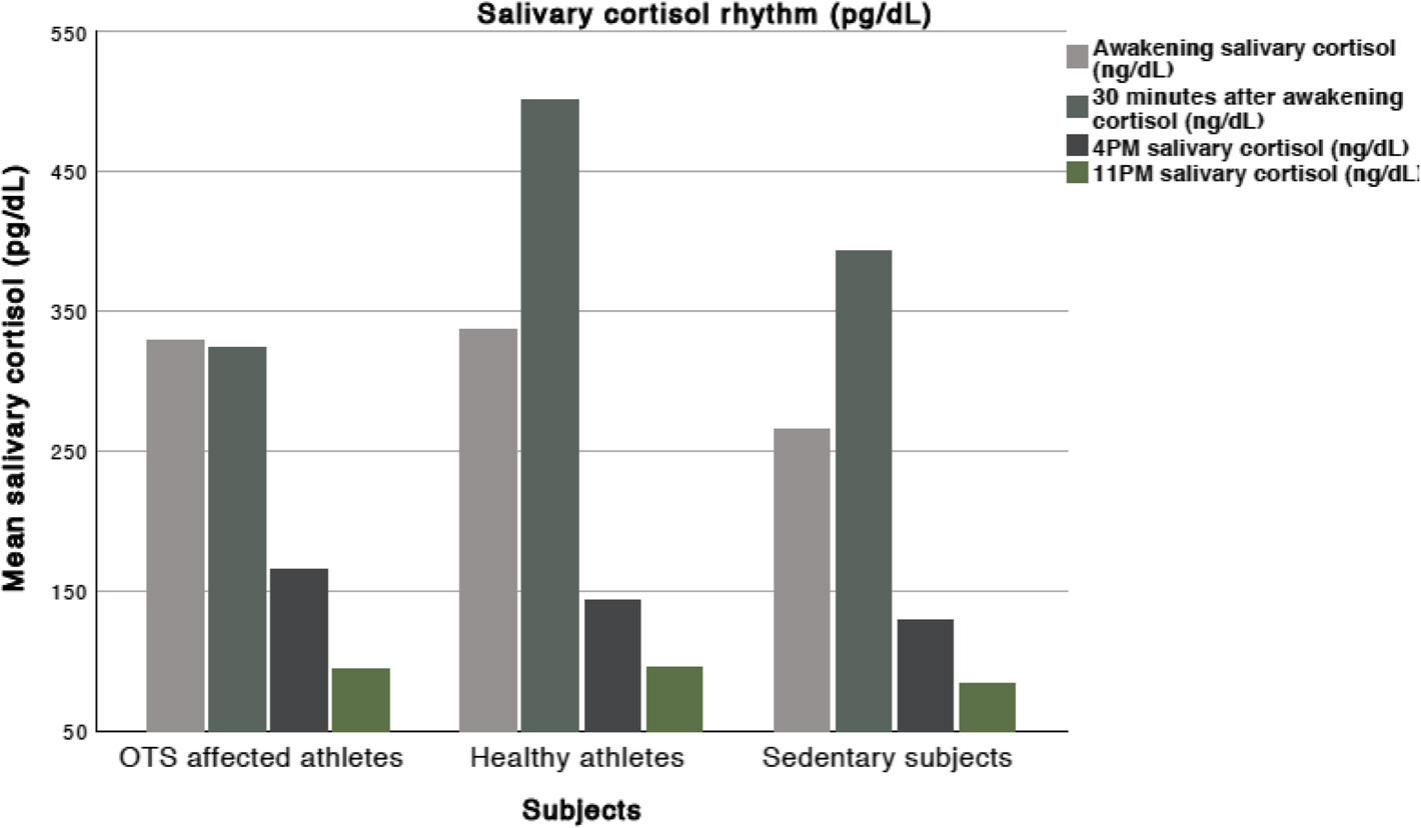
Salivary cortisol rhythm

SCR was assessed in 23 of the 25 ATL and in all OTS and NCS. SCR was similar among groups, although ATL presents exacerbated and significant elevation on 30 min after awakening compared to OTS and NCS (Table 5 and Fig. 4). Conversely, CAR, awakening, 4 PM, and 11 PM salivary cortisol levels were similar between OTS, ATL, and SED. Although CAR was higher in ATL, it did not achieve statistical significance.

**Table 5:** Mean and SD of salivary cortisol rhythm

Salivary cortisol (ng/dL)	OTS athletes (OTS) (*n* = 14)	Healthy athletes (ATL) (*n* = 25)	Non-active subjects (NCS) (*n* = 12)
Awakening	329 (± 222)	337 (± 131)	266 (± 149)
30′ after awakening	324^***^ (± 116)	500^&^ (± 168)	393 (± 149)
4 PM	166 (± 113)	144 (± 83)	130 (± 57)
11 PM	94 (± 39)	95 (± 38)	83 (± 11)
Cortisol awakening response (CAR) %	32.4	62.2	79.08

Differences between OTS and ATL: ***p < 0.005;

Differences between ATL and NCS: ^&^ *p* < 0.05

*SD* standard deviation

A cutoff of 370 ng/dL has a high accuracy (80%) but is not reliable to exclude or confirm OTS. Conversely, higher salivary cortisol levels (> 530 ng/dL) were not observed in any of the OTS and in only one NCS, whereas they were observed in majority of ATL (12 of 22; 59.1%), and therefore we suggest that a cutoff of > 530 ng/dL may be a tool to reinforce the exclusion of OTS (Fig. 4).

## Discussion

### Results

This study examined the role of the HPA axis in OTS. To learn whether the differences in hormone responses between OTS and ATL resulted from a physiological adaptation to sports (which has not been analyzed in previous studies) or from dysfunctional OTS responses, we included two control groups, of healthy athletes and healthy non-active subjects. Overall, we found that OTS have blunted hormonal responses compared to ATL, but not to NCS, whereas ATL showed more exacerbated responses than NCS. It means that athletes affected by OTS do not disclose actual dysfunctional responses, as hormonal responses were not different from normal non-active control subjects, but nullifies the likely adaptive hormonal responses observed in healthy athletes, as healthy athletes disclosed different responses compared to sedentary control subjects, whereas the different responses compared to NCS were not observed when compared to OTS-affected subjects. This means that the different responses, a likely adaptive process observed in healthy athletes, are nullified when OTS is present.

While CST reflects the adrenal status, ITT discloses the integrity of the entire HPA axis. As the adrenals responded normally in the abovementioned ACTH stimulation test, any abnormality found at the ITT must be located centrally (either in the hypothalamus or in the pituitary). Moreover, as the ITT directly stimulates the glands of the HPA axis (whose pathway of stimulation is the hypothalamus, then the pituitary, and then the adrenal glands, in this order), any alterations in responses due to differences in signaling coming from the neuromuscular and cardiovascular systems are abolished. Finally, the response to hypoglycemia does not depend on sports capacity and conditioning, and therefore, impaired hormone responses to ITT show a truly impaired HPA axis.

Indeed, differences in cortisol and ACTH responses were observed between groups, suggesting differences in the HPA axis responsivity to stressful situations. However, instead of the presence of dysfunctional impaired responses in OTS, exacerbated and hastened responses were observed in ATL, compared to NCS and OTS, for both ACTH and cortisol, whereas basal levels were similar among groups. Basal cortisol and ACTH levels tended to be normal in OTS, as reported by previous studies [1, 3, 6, 9–12], although some studies reported either reduced [23] or increased levels [34]; contrariwise, stimulated cortisol and ACTH levels showed predominantly reduced levels [1, 3, 7–9], which is corroborated by us.

Despite the higher cortisol in the ATL during hypoglycemia, the valuable point of cortisol is actually 30 min after the hypoglycemia event, due to the delay between ACTH stimulation and consequent cortisol release. Cortisol levels were already higher during hypoglycemia likely to the fact that the reduction in glucose levels and consequent ACTH stimulation began minutes before the hypoglycemia event. Although ACTH was not statistically higher during hypoglycemia in the ATL, the mean and median of ACTH levels were substantially increased compared to OTS and NCS. The reduced ACTH/cortisol ratio observed in the OTS group 30 min after hypoglycemia reflects the blunted ACTH levels observed in the OTS-affected athletes at this time. However, a reduced ACTH/cortisol ratio does not provide additional information in regard to a supposed level of sensitivity of the adrenal glands to the ACTH stimulation, as ACTH levels cannot be correlated with the cortisol levels of the same time [13, 32].

If we followed the adrenal criteria of < 18 μg/dL as relative adrenal insufficiency [33], the majority of OTS and NCS would be considered as affected, although we modified the ITT protocol after the hypoglycemic episode to prevent severe hypoglycemia [37, 38]. Importantly, the relative adrenal insufficiency is only found during a stimulation test and does not bring about the typical risks of the classical and frank primary adrenal insufficiency that can be diagnosed by elevated basal serum ACTH levels and reduced basal serum cortisol levels [33].

We did not perform the 60 min cortisol after hypoglycemia as we initially performed with some athletes (*n* = 13) and cortisol levels did not disclose increased responses between 30 and 60 min.

Furthermore, previous studies hypothesized that OTS was characterized by alterations in the sensitivity of the HPA axis [1, 7–11]. Therefore, the ACTH/cortisol ratio would be altered in OTS-affected athletes. We found the ACTH/cortisol ratio basally, during hypoglycemia, and 30 min after hypoglycemia was similar among the three groups at all times. While our results seem to invalidate the theory regarding the hyposensitivity of the HPA axis in OTS, an altered ACTH/cortisol ratio does not indicate a difference in response in the HPA axis, as the process of releasing ACTH to stimulate cortisol release can take a few minutes. Therefore, the cortisol level at a certain time point does not reflect the ACTH level at that same moment.

The loss of circadian rhythm of the cortisol release can be a feature of fatigue-related disorders, as previously described [13–17], although causality of adrenal impairment has not been demonstrated. However, an intact cortisol rhythm was observed in all subjects, regardless of the group. Nonetheless, an exacerbated cortisol awakening increase was observed in ATL, although CAR was not statistically significant due to high variation of the results. However, if there were three times more subjects, with the same median and confidence interval, statistical significance would be achieved for the CAR. The significant difference was found at 30 min after awakening and seemed to be a reliable marker of sports conditioning, whose adaption showed to disappear when OTS is present.

### The Hormonal Deconditioning Effect of OTS

It has been previously proposed that hypersensitivity of the HPA axis (during the beginning of the overtraining state process) followed by a progression to insensitivity is involved in OTS [1, 7, 8]. However, our results suggest that the hyposensitive HPA axis is only observed when compared to healthy athletes. We found the cortisol response to hypoglycemia was significantly higher among healthy athletes than OTS and NCS. This shows that the improvement in HPA axis sensitivity in athletes is loss during OTS, and not that OTS presents any frank dysfunctions, as OTS discloses similar responses compared to the general population (i.e., non-active healthy subjects with same age, gender, and BMI). Furthermore, while cortisol response was observed 30 min after hypoglycemia in OTS, it is unlikely that any further increased cortisol levels (at any time after 30 min) would be sustained, as ACTH (the major stimulator of cortisol release) levels were reduced at 30 min after hypoglycemia at this group.

In summary, all the findings of the present study, including cortisol and ACTH responses to ITT and salivary cortisol 30 min after awakening, seemed to be positive adaptions to exercises in healthy athletes (in the absence of OTS) that helped improve sport-specific performance, and for some reason, this hormonal conditioning (which were the positive adaptions to exercises) was at least partially lost in OTS, which may explain the decrease in performance in OTS.

As healthy athletes disclosed optimized HPA axis responses to hypoglycemia compared to sedentary individuals, we suggest that there is a possible hyperresponsivity of the HPA axis in athletes (as an adaption or conditioning response) that can be expressed independently of exercise.

### Correlations Between Findings with Clinical Features of OTS

Besides fatigue and underperformance, a key feature of OTS is the inability to sustain a sport-specific performance capacity throughout an intensive training session [1–4, 39, 40]. OTS-affected athletes are usually able to perform normally in the beginning of the training load, but are not able to complete at the expected pace (i.e., OTS-affected subjects tend to fatigue too early compared to healthy athletes). We found a similar early increase in ACTH levels among all three groups, but an early fall in ACTH levels among OTS-affected athletes, with especially blunted ACTH levels at 30 min after hypoglycemia. These findings (normal early ACTH increase with late blunted levels) may at least partly explain the reduced time-to-fatigue found in athletes affected by OTS. Indeed, ACTH and cortisol are two fundamental hormones required to sustain performance and pace in any sport. (Thus, as the integrity of the HPA axis is required throughout training to maintain performance, a shortened response duration of the HPA axis in OTS-affected athletes would affect sustained performance).

### Suggestions of Cutoffs for Biochemical Diagnosis of OTS

While the number of subjects included in this study was small, we were able to suggest helpful cutoffs for the diagnosis or exclusion of OTS due to a number of factors. First, the number of subjects of the present study is significantly higher than previous studies [3]. Second, the diagnosis of OTS was strictly performed (without confounding bias), and therefore all 14 OTS presented real OTS. Finally, the hormone levels were markedly different between OTS and ATL, with few overlapping results in most findings. A summary of the possible cutoffs to help confirm or exclude OTS are described in Table 6.

**Table 6:** Proposed cutoffs for overtraining syndrome

	Suggested cutoffs	Practical application
Salivary cortisol (ng/dL) 30 min after awakening	> 530 ng/dL	Highly predictable of exclusion of OTS (93.9%).
	370 ng/dL	Highly accurate (80%), but unable to help diagnosis OTS
Serum cortisol (μg/dL) 30 min after hypoglycemia in ITT	> 20.5 μg/dL	High negative predictive value for OTS (100%)
	> 17.0 μg/dL	High positive predictive value for OTS, although not specific (28.6%)
	19.1 μg/dL	High accuracy (84.6%), but not precise for confirmation or exclusion of OTS
Cortisol increase during ITT (μg/dL)	> 9.5 μg/dL	100% specific to exclude OTS
Plasma ACTH (pg/mL) 30 min after hypoglycemia in ITT	> 106 pg/mL	High negative predictive value (92.9%) and highly accurate (80%)
ACTH increase during ITT (pg/mL)	< 35 pg/mL	80% accurate to distinguish OTS from ATL

### Final Discussion

In summary, to the best of our knowledge, this is the first study on the endocrine system in OTS to (1) demonstrate that there are intrinsic dysfunctions of the HPA axis response to a stress situation in OTS-affected athletes, compared to healthy athletes; (2) demonstrate that the hypothalamus and the pituitary, and not the adrenals, are likely responsible for the changes in the cortisol response in an independent way from exercise-induced stimulation; (3) show that salivary cortisol levels 30 min after awakening are blunted in OTS-affected subjects, which may be a reliable marker of OTS; (4) propose useful cutoffs of salivary cortisol, and cortisol and ACTH responses to ITT, as an additional tool to help exclude or confirm OTS; (5) demonstrate that over-trained athletes have a blunted optimized hormone response to *training stress*; and (6) *indicate* that reduced time-to-fatigue, a key feature of OTS, not yet fully understood, seems to be at least partly explained by the present findings.

The main limitations of the present study are as follows: (1) the small number of subjects, due to the excluded subjects when strict criteria was used (but still larger compared to previous studies) and (2) a high variability and the lack of normal distribution observed in ACTH responses, which typically occurs in tests with ACTH responses, but challenges the statistical interpretation.

Moreover, the presence of outliers reinforces the individuality of overtraining syndrome presentation in each athlete, which leads to distinct responses to different tests. Also, as all athletes were considered as OTS in this study (that is, the prolonged *overtraining* state), it is unclear whether the changes could be found earlier in OTS, in the functioning and non-functioning *overreaching s*tates.

## Conclusions

Whereas healthy athletes seem to present hormonal conditioning adaptions, those affected by overtraining seem to have impaired or maladapted hormonal conditioning, which may explain the decreased performance observed during OTS, as a sort of deconditioning process. Our findings allowed us to suggest useful cutoff values for the 30 min after awakening cortisol, cortisol and ACTH 30 min after hypoglycemia, and cortisol and ACTH increase during the ITT to help exclude or confirm diagnosis of OTS. Despite these unprecedented findings, further studies are recommended to confirm our results.

## Abbreviations

ACTH: Adrenocorticotropic hormone
ATL: Healthy athletes group
CBG: Cortisol-binding globulin
CST: Cosyntropin stimulation test
EROS: Endocrine and Metabolic Responses on Overtraining Syndrome
ITT: Insulin tolerance test
NCS: Normal control subjects
OTS: Overtraining syndrome
SCR: Salivary cortisol rhythm

## References

[1] Meeusen R, Duclos M, Foster C, Fry A, Gleeson M, Nieman D, Raglin J, Rietjens G, Steinacker J, Urhausen A, European College of Sport Science; American College of Sports Medicine (2013). Prevention, diagnosis, and treatment of the overtraining syndrome: joint consensus statement of the European College of Sport Science and the American College of Sports Medicine. Med Sci Sports Exerc.

[2] Le Meur Y, Hausswirth C, Natta F (2013). A multidisciplinary approach to overreaching detection in endurance trained athletes. J Appl Physiol.

[3] Nederhof E, Zwerver J, Brink M, Meeusen R, Lemmink K (2008). Different diagnostic tools in nonfunctional overreaching. Int J Sports Med.

[4] Lehmann M, Foster C, Keul J (1993). Overtraining in endurance athletes: a brief review. Med Sci Sports Exerc.

[5] Cadegiani FA, Kater CE (2017). Hormonal aspects of overtraining syndrome: a systematic review. BMC Sports Sci Med Rehabil.

[6] Thiel C, Vogt L, Bürklein M, Rosenhagen A, Hübscher M, Banzer W (2011). Functional overreaching during preparation training of elite tennis professionals. J Hum Kinet.

[7] Meeusen R, Nederhof E, Buyse L, Roelands B, De Schutter G, Piacentini MF (2010). Diagnosing overtraining in athletes using the two-bout exercise protocol. Br J Sports Med.

[8] Meeusen R, Piacentini MF, Busschaert B, Buyse L, De Schutter G, Stray-Gundersen J (2004). Hormonal responses in athletes: the use of a two bout exercise protocol to detect subtle differences in (over)training status. Eur J Appl Physiol.

[9] Urhausen A, Gabriel HH, Kindermann W (1998). Impaired pituitary hormonal response to exhaustive exercise in overtrained endurance athletes. Med Sci Sports Exerc.

[10] Barron G, Noakes T, Levy W, Smidt C, Millar R (1985). Hypothalamic dysfunction in overtrained athletes. J Clin Endocrinol Metab.

[11] Gold P (1993). Immobolisation stress rapidly decreases hypothalamic corticotropin-releasing hormone secretion in vitro in the male 344/N Fischer rat. Life Sci.

[12] Duclos M, Corcuff J-B, Arsac L (1998). Corticotroph axis sensitivity after exercise in endurance-trained athletes. Clin Endocrinol.

[13] Cadegiani FA, Kater CE (2016). Adrenal fatigue does not exist: a systematic review. BMC Endocr Disord.

[14] Ryan R, Booth S, Spathis A, Mollart S, Clow A (2016). Use of salivary diurnal cortisol as an outcome measure in randomised controlled trials: a systematic review. Ann Behav Med.

[15] Stalder T, Kirschbaum C, Kudielka BM (2016). Assessment of the cortisol awakening response: expert consensus guidelines. Psychoneuroendocrinology.

[16] Oosterholt BG, Maes JH, Van der Linden D, Verbraak MJ, Kompier MA (2015). Burnout and cortisol: evidence for a lower cortisol awakening response in both clinical and non-clinical burnout. J Psychosom Res.

[17] Powell DJ, Liossi C, Moss-Morris R, Schlotz W (2013). Unstimulated cortisol secretory activity in everyday life and its relationship with fatigue and chronic fatigue syndrome: a systematic review and subset meta-analysis. Psychoneuroendocrinology.

[18] Slivka DR, Hailes WS, Cuddy JS, Ruby BC (2010). Effects of 21 days of intensified training on markers of overtraining. J Strength Cond Res..

[19] Rietjens GJ, Kuipers H, Adam JJ (2005). Physiological, biochemical and psychological markers of strenuous training-induced fatigue. Int J Sports Med.

[20] Coutts AJ, Reaburn P, Piva TJ, Rowsell GJ (2007). Monitoring for overreaching in rugby league players. Eur J Appl Physiol.

[21] Schmikli SL, de Vries WR, Brink MS, Backx FJ (2012). Monitoring performance, pituitary-adrenal hormones and mood profiles: how to diagnose non-functional over-reaching in male elite junior soccer players. Br J Sports Med.

[22] Tanskanen MM, Kyröläinen H, Uusitalo AL, Huovinen J, Nissilä J, Kinnunen H, Atalay M, Häkkinen K (2011). Serum sex hormone-binding globulin and cortisol concentrations are associated with overreaching during strenuous military training. J Strength Cond Res.

[23] Kraemer WJ, French DN, Paxton NJ (2004). Changes in exercise performance and hormonal concentrations over a big ten soccer season in starters and nonstarters. J Strength Cond Res.

[24] Hug M, Mullis PE, Vogt M, Ventura N, Hoppeler H (2003). Training modalities: over-reaching and over-training in athletes, including a study of the role of hormones. Best Pract Res Clin Endocrinol Metab..

[25] Steinacker JM, Lormes W, Kellmann M (2000). Training of junior rowers before world championships. Effects on performance, mood state and selected hormonal and metabolic responses. J Sports Med Phys Fitness.

[26] Fry AC, Kraemer WJ, Ramsey LT (1998). Pituitary-adrenal-gonadal responses to high-intensity resistance exercise overtraining. J Appl Physiol (1985).

[27] Fry RW, Morton AR, Garcia-Webb P, Crawford GP, Keast D (1992). Biological responses to overload training in endurance sports. Eur J Appl Physiol Occup Physiol.

[28] Lehmann M, Gastmann U, Petersen KG, Bachl SA, Khalaf AN, Fischer S, Keul J (1992). Training-overtraining: performance, and hormone levels, after a defined increase in training volume versus intensity in experienced middle- and long-distance runners. Br J Sports Med.

[29] O'Connor PJ, Morgan WP, Raglin JS, Barksdale CM, Kalin NH (1989). Mood state and salivary cortisol levels following overtraining in female swimmers. Psychoneuroendocrinology.

[30] Barron LJ, Noakes TD, Levy W, Smith C, Millar RP (1985). Hypothalamic dysfunction in overtrained athletes. J Clin Endocrinol Metab.

[31] Cadegiani FA, Kater CE. Rationale, design, material, methods, subject selection and baseline characteristics of the Endocrine and Metabolic Responses on Overtraining Syndrome (EROS) study. Available at: https://osf.io/bhpq9/.

[32] Ospina NS, Al Nofal A, Bancos I, Javed A, Benkhadra K, Kapoor E (2016). ACTH stimulation tests for the diagnosis of adrenal insufficiency: systematic review and meta-analysis. J Clin Endocrinol Metab.

[33] Bornstein SR, Allolio B, Arlt W, Barthel A, Don-Wauchope A, Hammer GD (2016). Diagnosis and treatment of primary adrenal insufficiency: an endocrine society clinical practice guideline. J Clin Endocrinol Metab.

[34] Roberts AC, McClure RD, Weiner RI, Brooks GA (1993). Overtraining affects male reproductive status. Fertil Steril.

[35] Bae YJ, Kratzsch J (2015). Corticosteroid-binding globulin: modulating mechanisms of bioavailability of cortisol and its clinical implications. Best Pract Res Clin Endocrinol Metab.

[36] Perogamyros I, Aarons L, Miller AG, Trainer PJ, Ray DW (2011). Corticosteroid-binding globulin regulates cortisol pharmacokinetics. Clin Endocrinol.

[37] Brooks K, Carter J. Overtraining, exercise and adrenal insufficiency. J Nov Physiother. 2013;3(125). 10.4172/2165-7025.1000125 23667795

[38] Angeli A, Minetto M, Dovio A, Paccotti P (2004). The overtraining syndrome in athletes: a stress-related disorder. J Endocrinol Investig.

[39] Urhausen A, Gabriel H, Kindermann W (1995). Blood hormones as markers of training stress and overtraining. Sports Med.

[40] Budgett R, Newsholme E, Lehmann M (2000). Redefining the overtraining syndrome as the unexplained underperformance syndrome. Br J Sports Med.

